# Reduced Susceptibility of a *Biomphalaria tenagophila* Population to *Schistosoma mansoni* after Introducing the Resistant Taim/RS Strain of *B. tenagophila* into Herivelton Martins Stream

**DOI:** 10.1371/journal.pone.0099573

**Published:** 2014-06-18

**Authors:** Daisymara Priscila de Almeida Marques, Florence Mara Rosa, Engels Maciel, Deborah Negrão-Corrêa, Horácio Manuel Santana Teles, Roberta Lima Caldeira, Liana Konovaloff Jannotti-Passos, Paulo Marcos Zech Coelho

**Affiliations:** 1 Laboratory of Schistosomiasis, Research Center René Rachou/FIOCRUZ, Belo Horizonte, Minas Gerais, Brazil; 2 Laboratory of Parasitology, Institute of Biological Sciences, Federal University of Juiz de Fora, São Pedro, Minas Gerais, Brazil; 3 Chácara Santa Inês, Bananal, São Paulo, Brazil; 4 Laboratory of Schistosomiasis, Institute of Biological Sciences, Federal University of Minas Gerais, Belo Horizonte, Brazil; 5 Superintendência de Controle de Endemias do Estado de São Paulo/SUCEN, São Paulo, Brazil; 6 Laboratory of Helminthology and Medical Malacology, Research Center René Rachou/FIOCRUZ, Belo Horizonte, Minas Gerais, Brazil; 7 Mollusk Room Dr. Lobato Paraense, Research Center René Rachou/FIOCRUZ, Belo Horizonte, Minas Gerais, Brazil; AC Camargo Cancer Hospital, Brazil

## Abstract

Studies performed in the last 30 years demonstrated that a strain of *B. tenagophila* from the Taim Biological Reserve is completely resistant to *Schistosoma mansoni* infection. This resistance to parasite infection is a dominant characteristic during crossbreeding with susceptible *B. tenagophila* strains. These experiments also identified a 350 bp molecular marker that is exclusive to the Taim strain and does not occur in other geographic strains of this snail species. The Taim strain (Taim/RS) of *Biomphalaria tenagophila* was bred on a large scale, physically marked and introduced into a stream in which previous malacological analyses had revealed the presence of only parasite-susceptible *B. tenagophila*. Samples of offspring captured 4, 11 and 14 months after the introduction of the Taim strain were examined, and the susceptibility of the snails to *S. mansoni* infection dropped from 38.6–26.5% to 2.1% during the 14 months after the introduction of the Taim snail strain. A significant correlation was also observed between the absence of infection and the identification of the Taim molecular marker. These results demonstrate that the genetic marker from the Taim strain was successfully introduced into the wild snail population. In addition, a significant relationship exists between the marker and resistance to infection.

## Introduction

In Brazil, *Biomphalaria tenagophila* (d’Orbigny, 1835) is the second most important species for schistosomiasis transmission and is responsible for transmission of this disease in large areas of the state of São Paulo [1] and in isolated foci in the states of Minas Gerais, Rio de Janeiro and Santa Catarina [2]–[4].

*B. tenagophila* strains from different geographic areas exhibit varying rates of infection after exposure to *Schistosoma mansoni* [5]–[7]. Although many populations of *B. tenagophila* are susceptible to the parasite, a particular population collected at the Taim Biological Reserve in the State of Rio Grande do Sul, Brazil is completely resistant to *S. mansoni. B. tenagophila* Taim has been investigated by researchers for several decades [8]–[23]. The results of these studies demonstrated that *B. tenagophila* Taim is resistant to a number of different *S. mansoni* strains. These studies also demonstrated that this resistance is due to an efficient internal defense system [12], [16], [18]. Experimental infections revealed that after miracidium penetration, an intense cellular reaction occurs; this reaction is driven primarily by hemocytes and destroys the parasite during the early hours of infection [21], [22].

Crossbreeding experiments between *B. tenagophila* Taim, which is resistant, and *B. tenagophila* from Joinville, which is susceptible, demonstrated that resistance to *S. mansoni in B. tenagophila* is a dominant characteristic [14]. The transmission of the resistance phenotype of *B. tenagophila* (Taim) was maintained in the susceptible population, regardless of the presence of parasite pressure [23]. Even in experimental groups in which the proportion of resistant snails was lower than the proportion of susceptible snails, resistance was successfully transmitted to individuals in the F_1_, F_2_ and F_3_ generations [23]. The same study [23] also demonstrated that *S. mansoni* exerted negative selective pressure on susceptible populations. This result is not surprising because *S. mansoni* infection is highly virulent in the snail host, causing high host mortality rates and partial host castration; thus, this infection reduces snail fitness [24], [25]. In addition, the resistant snail strain reproduced better than the susceptible strain even in the absence of the parasite. Increased reproductive effort was also reported in *B. glabrata* after exposure to *S. mansoni* that failed to develop into an infection, suggesting that this strategy is important for fecundity compensation in response to increasing parasite pressure and subsequent increasing mortality and morbidity risk [26].

Polymerase chain reaction-restriction fragment length polymorphism (PCR-RFLP) analysis of the internal transcribed spacer region (ITS) of ribosomal DNA using the restriction enzyme Ddel verified that the *B. tenagophila* Taim population exhibits a molecular profile with three ITS fragments (i.e., fragments of 800, 470 and 350 bp); other *B. tenagophila* populations in Brazil only exhibit the first two bands [17]. Thus, the 350 bp fragment is typical of the Taim snail strain. Although the Taim molecular marker is only a fragment of the ribosomal DNA and likely plays no role in the resistance of the snails to infection, this fragment exhibits a dominant genotype [27] and therefore constitutes a valuable molecular marker for the Taim strain in both laboratory and field studies.

Based on these data, our research group suggested that the Taim strain represents a novel tool for the biological control of schistosomiasis transmission. The methodology for exploiting this tool includes introducing a large number of *B. tenagophila* (Taim/RS) snails into water bodies where this species is the only transmitter of *S. mansoni* [19]. The introduction of the Taim lineage can be monitored by identifying the 350 bp molecular marker in the offspring that result from crossbreeding between resistant *B. tenagophila* snails (i.e., introduced snails) and susceptible snails (i.e., local snails). The preliminary results obtained in previous field studies have been promising, although it was not possible to evaluate the impact of the Taim strain on the susceptibility of the local snails in these analyses [19]. Therefore, the main purpose of the present work was to analyze susceptibility to infection and the presence of the marker in the descendants of crosses between Taim and local *B. tenagophila* snails collected 4, 11 and 14 months after the introduction of the Taim strain into a stream in the municipality of Bananal in the state of São Paulo, Brazil.

## Materials and Methods

### Characterization of the Study Area

This study was performed in the Herivelton Martins stream (latitude 22° 43′ 706″ and longitude 44° 21′409″), which is located in the neighborhood of Santo Antonio do Retiro in the municipality of Bananal in the state of São Paulo, Brazil. The stream runs for a length of 42 m, with an average width of 1.5 m and a depth of up to 1.5 m, before debouching into another stream with a larger volume. Previous studies indicated that *B. tenagophila* is the only *Biomphalaria* species present in this stream and in all water samples collected in the municipality of Bananal [28], [29].

### Introduction and Assessment of *B. tenagophila* Taim in the Study Stream

Large-scale breeding of *B. tenagophila* Taim was performed in a mollusk facility at the Chácara Santa Inês–Vila Bom Jardim, which is located near the experimental field in Bananal/SP, according to procedures described by Rosa et al. [30].

A total of 800 *B. tenagophila* snails (Taim strain) that were 9 to 12 mm in diameter were physically marked with a small hole (1 mm diameter) on their side, near the top opening of the shell. A nylon thread was subsequently inserted into this hole, and a knot was made, forming a small ring. This procedure allowed easy differentiation of the local snails (i.e., unmarked snails) from the introduced resistant population (i.e., marked snails). After marking, the resistant *B. tenagophila* specimens were introduced into the superior portion of the Herivelton Martins stream. Before the introduction of marked *B. tenagophila* Taim, a sample of the local snail strain was collected for further studies of parasite susceptibility.

Fifteen days after the introduction of the snails, snails were collected from the stream and examined for the presence of the physical markers on their shells. The total number of marked and unmarked snails was counted within the collected population, and these numbers were used to assess the proportion of introduced resistant snails (i.e., marked snails) in relation to the local snails (i.e., unmarked snails) [31]. After the examination, the marked and unmarked snails were returned to the stream.

After this initial analysis that focused on the introduced population, three additional snail collections were conducted in the stream; these collections were performed 4, 11 and 14 months after the introduction of the Taim snails. Under the local environmental conditions, 3–4 snail generations (i.e., from egg to egg-laying adult) were expected to occur from the time of Taim snail introduction until the time of the last collection (i.e., 14 months later). During these three field collections, only offspring snails, which were characterized by a shell size less than 5 mm in diameter, were collected and examined in the laboratory. The collected specimens were individually exposed to artificial light for approximately 4 h to stimulate cercarial shedding [32]. After parasite examination, 50 snails from each collection period (i.e., 4, 11 and 14 months) were randomly selected for molecular analysis, as described below, and the remaining population was maintained at the Mollusk Room Lobato Paraense/CPqRR. The F1 descendants of the snails collected at each time point were used for *S. mansoni* infection susceptibility testing, as described below.

### Identification of the Taim Molecular Marker

As mentioned above, 50 snail specimens, which were collected 4, 11 and 14 months after the Taim introduction, were used for the molecular analysis. Total DNA was extracted from the cephalopodal region of each snail using the Wizard Genomic DNA purification kit (Promega Corporation, Madison, WI, USA) according to the manufacturer’s instructions, with some modifications [33].

The DNA extracted from each snail was analyzed via PCR. The entire ITS sequence was amplified using the primers ETTS2 (5′TAACAAGGTTTCCGTAGGTGAA3′) and ETTS1 (5′TGCTTAAGTTCAGCGGGT3′), which were anchored in the conserved extremities of the 18S and 28S ribosomal genes, respectively [34]. The PCR amplifications were performed in a volume of 10 µl that contained the following components: 1 to 10 ng of template DNA, 10 mM Tris-HC1 (pH 8.5), 200 µM of each dNTP, 1.5 mM MgC12, 0.5 U of Taq DNA polymerase, 50 mM KC1 and 1.0 pmol of each primer. The mixtures were covered with a drop of mineral oil and subjected to the following cycling program: an initial denaturation step for 3 min at 95°C, followed by 32 cycles of annealing at 54°C for 1 min, extension at 72°C for 2 min, and denaturation at 95°C for 45 s and a final extension step at 72°C for 5 min. A negative control (i.e., no template DNA) was included in all experiments. Three microliters of the amplification products were visualized on 6% silver-stained polyacrylamide gels to determine the quality of amplification.

The approximately 1,300 bp fragment that was obtained using the primers ETTS_1_ and ETTS_2_ was digested with the enzyme Ddel. Briefly, the remaining amplified material (7 µl) was diluted in 63 µl of sterile water and 10 µl of the diluted PCR product, 0.3 µl (4–8 units) of the Ddel enzyme (Promega) and 1 µl of the enzyme buffer (Promega) were included in each digestion. The final volume of the reaction was 11.3 µl, and the digestion was incubated for 3.5 h at 37°C. The obtained restriction profiles were visualized on a 6% polyacrylamide gel that was silver-stained. The expected species-specific profile for *B. tenagophila* consists of two fragments (i.e., 800 and 470 bp). For Taim lineage snails, an additional fragment of 350 bp is also expected.

### Susceptibility of the Snails to *Schistosoma mansoni*

The SJ strain of *S. mansoni* was used in this experiment. This parasite strain was isolated from *B. tenagophila* collected in São José dos Campos/SP in the Rio Paraíba Valley and has been maintained in the Mollusk Room Lobato Paraense/CPqRR for over 40 years in hamsters (*Mesocricetus auratus*) and *B. tenagophila*, with sporadic passages in *Biomphalaria glabrata*.

For the susceptibility test, the young snails collected in Herivelton Martins stream prior to the introduction of the resistant snail strain and 4, 11 and 14 months after the introduction of the resistant snail strain were maintained in a separate aquarium to grow and produce eggs. The egg masses collected from each group were separated and grown until they reached 6–8 mm in diameter. At this stage, the snails were individually exposed to 25 *S. mansoni* miracidia of the SJ strain. Specimens of highly susceptible *B. glabrata,* which were provided by the Mollusk Room Lobato Paraense/CPqRR, were infected with the same sample of *S. mansoni* miracidia; these snails represented the positive control group.

Two tests of susceptibility to *S. mansoni* infection were performed. In the first test, the offspring obtained from eggs laid by the snails collected in the Herivelton Martins stream prior to introduction and 4 months post-introduction were exposed to the parasite. The second susceptibility test was performed using offspring snails isolated from the local snail strain prior to the Taim introduction and from the snails collected 11 and 14 months post-introduction. Beginning 26 days after infection, each snail was examined weekly for a 2-month period to detect cercarial shedding.

The infected and uninfected snails from different experimental groups were later subjected to PCR-RFLP analysis, as described above, to detect correlations between resistance and the presence of the 350 bp molecular marker.

### Statistical Analysis

To identify possible associations or differences among the analyzed groups, Pearson’s chi-square test, which is suitable for comparisons of proportions, was used. When data were present at low frequencies of less than 5 events, Fisher’s exact test was utilized. In all analyses, a significance level of 5% was used. Calculations were performed using the statistical software StatXact version 6.0 and Microsoft Office Excel 2007.

### Licenses and Authorizations

This experiment was approved by IBAMA (i.e., the Brazilian Institute of Environment and Renewable Natural Resources), which belongs to the Ministry of Environment. The scientific project was approved by IBAMA on December 29^th^, 2005 (number 02027.00687/2006-68) and by the Scientific Committee of SUCEN (Superintendencia de Controle de Endemias do Estado de São Paulo) (process number 359/2009).

## Results

### Estimation of Population Density

At 15 days post-introduction, the proportions of the *B. tenagophila* local population and the *B. tenagophila* Taim population present in the Herivelton Martins stream were estimated. A total of 237 snails were recovered; 89 of these snails were physically marked as the Taim population, and the remaining 148 snails represented the local strain. The proportion of the local population (62.5%) was significantly higher than that of *B. tenagophila* Taim (37.5%) (*p*<0.04).

### Parasitological and Molecular Results for Snails Collected 4, 11 and 14 Months after the Introduction

Throughout the evaluation period, 773 young snails were collected; none of these snails were observed to be infected with *S. mansoni* cercariae. However, some specimens were infected with xiphidiocercariae from another trematode species.

After parasitological examination, 50 of the young snails from each collection period were randomly subjected to PCR-RFLP to identify the 350 bp molecular marker indicative of the *B. tenagophila* Taim population. After the introduction of the resistant snails, the proportions of young snails that contained the molecular marker were as follows: 37.1% after 4 months, 35.7% after 11 months and 60% after 14 months. The data demonstrated that the proportion of snails carrying the Taim molecular marker was significantly higher in individuals collected at the 14-month time point (*p*<0.05).

**Figure 1:**
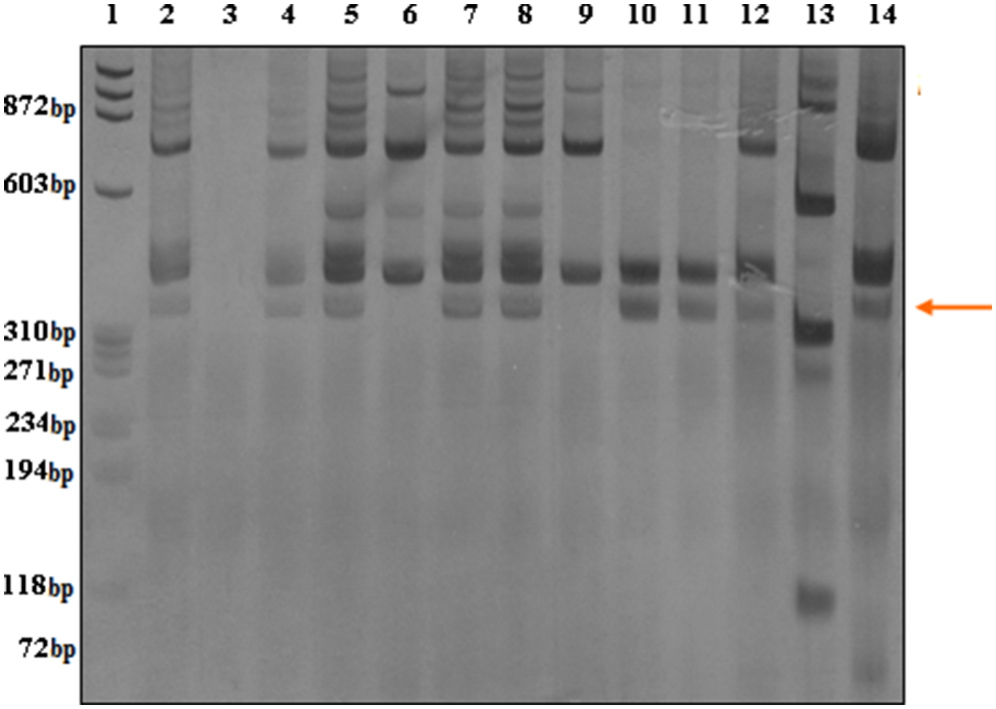
Silver-stained 6% polyacrylamide gel showing RFLP profiles. Lane 1: molecular size markers (PhiX 174); Lane 3: negative control (i.e., no template DNA); Lane 13: pool of *S. mansoni* cercariae; Lane 14: *B. tenagophila* Taim; Lanes 6 and 9: profile of the local strain of *B. tenagophila*; Lanes 2, 4–5, 7–8 and 10–12: profiles of *B. tenagophila* collected 14 months after Taim introduction (i.e., containing the 350 bp marker).

As illustrated in Fig. 1, the 350 bp marker was detected in a large proportion of the descendants collected 14 months post-introduction (lanes 2, 4, 5, 7, 8, 10, 11, and 12). These individuals also exhibited a profile similar to that of the Taim lineage (lane 14).

### Susceptibility Experiments

The data presented in Table 1 demonstrate that a progressive reduction of *S. mansoni* susceptibility occurred among the offspring of the snails collected from the stream after the introduction of the parasite-resistant Taim strain. In the first challenge with the SJ strain of *S. mansoni*, 38.6% of the of the snail population collected prior to the Taim introduction were susceptible to the parasite. In contrast, only 14.9% of the offspring from the population collected 4 months post-introduction shed cercariae. The group of snails collected after 4 months exhibited a significantly reduced infection rate (*p* = 0.003).

**Table 1:** Susceptibility of *B. tenagophila* collected before and after the introduction of the resistant lineage to infection after exposure to the SJ strain of *S. mansoni*.

	Snails exposed (n)	Survival (n)	Uninfected (negative) (n)	Infected (positive) (n)	Infection rate %	*P value*
**1^st^ Test**						
Before	70	57	35	22	38.6	0.003*
4 months (PI)	70	67	57	10	14.9	
**2^nd^ Test**						
Before	50	49	36	13	26.5	
11 months (PI)	50	44	41	3	6.8	0.012*
14 months (PI)	50	48	47	1	2.1	0.001*

PI: post-introduction.

*Significant differences between *B. tenagophila* collected before and after the introduction of the resistant lineage (*p*<0.05). Pearson’s chi-square test which is suitable for comparisons between proportions, and Fisher’s exact test were used at frequencies lower than 5.

In the second trial, the *S. mansoni* infection rate among the offspring of the original snail population prior to the Taim introduction was 26.5%. This value was significantly reduced to 6.8% and 2.1% among the snail populations descendant from the specimens collected 11 months (*p* = 0.0012) and 14 months post-introduction of the Taim snails (*p* = 0.001), respectively.

In both experiments, *B. glabrata* snails were used as control groups, and the experimental infection rate was 100% when these snails were exposed to miracidia of the SJ strain of *S. mansoni*.

### Identification of the 350 bp Molecular Marker in Uninfected and Infected Snails

To verify the proportions of infected (i.e., positive) and uninfected (i.e., negative) snails that carried the molecular marker indicative of the Taim strain (i.e., the 350 bp DNA fragment), a sample of the snails that had been exposed to infection was subjected to PCR-RFLP analysis. The data presented in Table 2 demonstrate that only 1 (2.3%) of the 43 snails harboring the Taim molecular marker was infected with *S. mansoni*. In contrast, 13 (30.2%) of the 43 snails lacking this marker were infected. The results revealed a correlation between the presence of the *B. tenagophila* Taim molecular marker in the offspring and resistance to infection by *S. mansoni.*

**Table 2:** Presence of the 350/resistance to *S. mansoni*. (Total: challenged snails = 86; infected snails = 14).

	Uninfected (%)	Infected (%)	Total
With 350 bp molecular marker	42 (98.7)	1 (2.3)	43
Without 350 bp molecular marker	30 (69.8)	13 (30.2)	43

Fisher’s Exact Test – *p*<0.001.

## Discussion

The transmission of *S. mansoni* resistance from *B. tenagophila* Taim to susceptible snail strains was tested in the municipality of Bananal in the state of São Paulo, Brazil. This municipality was chosen because the area had been studied previously and because well-documented data regarding schistosomiasis transmission in this area were available. These data clearly indicated that *B. tenagophila* is the only species of mollusk responsible for the transmission of the disease in this area [28], [29]. Adopted control measures, such as the treatment of people infected with *S. mansoni,* the use of molluscicides and the performance of basic sanitation services by technicians at residences in the urban area of the municipality, have reduced the schistosomiasis prevalence to below 1%. However, some human carriers of the parasite persist in the area, maintaining the possibility of eventual reactivation of transmission foci due to the presence of susceptible mollusks. Since 2002, no reports of naturally infected specimens of *B. tenagophila* in water samples collected in Bananal have been made, but experimental infections demonstrate that these populations of *B. tenagophila* can exhibit variable infection rates that range between 26.5 and 38.6% after exposure to the SJ strain of *S. mansoni* under laboratory conditions.

In this study, we evaluated the impact of *B. tenagophila* Taim on the susceptibility of local strains to *S. mansoni* after crossbreeding. Based on the assumption that snails of the genus *Biomphalaria* favor crossbreeding [35] and on evidence demonstrating that the *S. mansoni* resistance of Taim snails is dominant, we expected that the genetic signature of the Taim strain, including its resistance genes, would be transmitted to the local snails over time [14].

Previous studies demonstrated the success of this approach after the introduction of resistant snails [19], [36]. Our results indicate that even when *B. tenagophila* Taim (i.e., the resistant snail strain) is present at lower proportions (37.5%) than the local strain, crossbreeding and transmission of *S. mansoni* resistance to descendants occurred. As mentioned above, the 350 bp molecular marker indicative of the Taim strain was detected in 37.1%, 35% and 60% of the snails analyzed 4, 11 and 14 months after the introduction of Taim snails to the area, respectively. This molecular marker is a fragment of a spacer region of snail ribosomal DNA (i.e., an internal transcribed region) that is likely not linked to resistance against *S. mansoni* infection [19], [27]. However, the results clearly demonstrated that the Taim strain genome was successfully introduced and propagated among the local snail population and that this introduction was associated with increased resistance to *S. mansoni* infection. Despite the short length of the Herivelton Martins stream, the results of the present study provide information that supports the implementation of new experiments to validate this approach in other environments, using *B. tenagophila* as a unique transmitter species. If the results obtained in new areas corroborate our current data, the addition of parasite-resistant snails may represent a new tool for controlling schistosomiasis in endemic Brazilian areas populated by *B. tenagophila.*
